# A probabilistic approach to dispersal in spatially explicit meta-populations

**DOI:** 10.1038/s41598-020-79162-9

**Published:** 2020-12-17

**Authors:** Rajat Karnatak, Sabine Wollrab

**Affiliations:** 1 Leibniz-Institute of Freshwater Ecology and Inland Fisheries, Müggelseedamnn 310, 12587 Berlin, Germany; 2 Berlin-Brandenburg Institute of Advanced Biodiversity Research (BBIB), 14195, Berlin Germany

**Keywords:** Ecology, Ecological modelling, Theoretical ecology

## Abstract

Meta-population and -community models have extended our understanding regarding the influence of habitat distribution, local patch dynamics, and dispersal on species distribution patterns. Currently, theoretical insights on spatial distribution patterns are limited by the dominant use of deterministic approaches for modeling species dispersal. In this work, we introduce a probabilistic, network-based framework to describe species dispersal by considering inter-patch connections as network-determined probabilistic events. We highlight important differences between a deterministic approach and our dispersal formalism. Exemplified for a meta-population, our results indicate that the proposed scheme provides a realistic relationship between dispersal rate and extinction thresholds. Furthermore, it enables us to investigate the influence of patch density on meta-population persistence and provides insight on the effects of probabilistic dispersal events on species persistence. Importantly, our formalism makes it possible to capture the transient nature of inter-patch connections, and can thereby provide short term predictions on species distribution, which might be highly relevant for projections on how climate and land use changes influence species distribution patterns.

## Introduction

One of the central aims of meta-population theory is to better understand how species distribution is determined by dispersal, spatial distribution of habitats, as well as local patch dynamics^1–3^. A first theoretical description of meta-populations—formed by a species sub-populations, distributed on a landscape consisting of a certain number of habitable patches and connected via species dispersal—was introduced by Levins^4^. Levins’ approach was formulated as a probability based species presence/absence model, for infinitely many, identical, and equally connected sub-populations. This dynamic model estimated the probabilities of patch occupancy based on the colonization and extinction rate parameters. More realistic extensions of Levins model have since been proposed which include (1) size structured patch populations with dynamical models to simulate the behavior of local populations at the patch level^5–7^, (2) finite size stochastic models^8–10^ to incorporate the effects of noise on system dynamics, and (3) finite, spatially realistic realizations of meta-population dynamics^11–13^. Consequently, these extensions have helped (1) to move beyond the basic presence/absence description of patch populations, (2) in replacing the deterministic extinction time thresholds by temporal distributions due to colonization–extinction stochasticity, and (3) have provided the ability to incorporate heterogeneous patch characteristics and connectivity in meta-populations, respectively.

Spatially realistic models have shown that, besides patch quality, the spatial network structure (topology) has strong effects on patch occupancy^14^, species persistence^15^ and species recovery^16^. In addition to network topology, predictions from spatial realistic models critically depend on the implementation of the dispersal process and its dependency on patch characteristics, as well as species specific dispersal abilities. Different modeling approaches have implemented species dispersal in various ways, for instance, implicit or two patch models assume an all-to-all connected system^17–21^, so effectively patch location and between patch distance are inconsequential for dispersal. In spatially explicit multi-patch models, dispersal has to come with certain assumptions on how it depends on patch distance. The widely used spatially realistic approach by Hanski et al.^11,12^ describes dispersal of a dynamic meta-population with an exponential decay with distance from a source patch in all directions. In essence, these existing approaches model dispersal as a deterministic process, even if they take into account prevalent short term changes in the connectivity^22^. These deterministic conjectures on dispersal might work in specific instances, namely, (1) a spatially implicit all-to-all connected system can be a useful approximation to model the average system behavior for a sufficiently patch–dense meta-population, and (2) an exponentially decaying uniform dispersal approach can model overall dispersal patterns in a long time limit of a spatially explicit meta-population. However, treating dispersal from a deterministic perspective completely ignores the inherent stochasticity^23–25^ of the process—where an individual ends up in one out of all possible patches it could possibly reach—thereby ignoring the associated variability, especially on shorter time scales. Drivers of variability include changes in environmental flows, wind speed, and direction for instance, which can be quite dynamic on relatively shorter time scales—days or weeks compared to seasonal or annual—and can affect the dispersal patterns of species^26^. The variability with shorter time scales might also be highly relevant for projections on changes in species distribution patterns due to climate change and changes in land use patterns, influencing habitat suitability and distribution, as a basis for successful mitigation strategies. Furthermore, there is also the possibility for an individual of not reaching any patch successfully as losses generally occur during dispersal. Dispersal between connected patches will also entail a loss of individuals during transport due to the local patch environment and species characteristics—since no natural process is fully efficient—and this fact is often ignored by modeling studies.

To overcome the limitations of existing deterministic approaches with respect to spatially realistic dispersal, we introduce a spatially explicit, network-based framework which enables us to treat dispersal from a more general, probabilistic perspective. Networks have found substantial application in food-web ecology^27–29^, and network-based studies have also started to explore the dynamical aspects of meta-populations/communities^30,31^. It is indeed quite natural to consider meta-populations/communities as networks: regarding patches as nodes and a dispersal event between patches as a connection. In the proposed approach, an underlying distance matrix defines the overall landscape via inter-patch distances. This covers all potential dispersal routes of the network, while only a subset of these are realized via actual dispersal, represented by a connectivity/adjacency/dispersal matrix. Dispersal itself is implemented as a probable event, depending upon species dispersal ability, patch size and inter-patch distance, and is implemented via a directed network which captures directional dispersal, i.e. dispersal from a patch to another patch in the system does not imply reverse dispersal between them. In contrast to existing studies, the proposed framework allows to cover a broad range of connectivity scenarios, from all-to-all connected, to spatially explicit, and even captures the effects of transient and dynamic inter-patch connectivity over time. This framework enables us to more realistically simulate connectivity on shorter time scales, while at the same time providing average connectivity characteristics in the long time limit, which are consistent with existing all-to-all connected (for higher patch densities) or other deterministic spatially realistic dispersal approaches. To illustrate the main features of our network-based approach, we contrast predictions from all-to-all connected to fixed and dynamic network topologies derived via the introduced probabilistic dispersal perspective. While we limit our investigations in this paper to meta-populations, this approach can easily be extended to meta-communities.

## Model

To introduce our approach, we modify and study a system of equations based on a meta-community model introduced by Wang and Loreau (WL)^18^. We extend their two-patch system to a spatially explicit multi-patch model by incorporating a directed network into the set of equations. Additionally, we take into account losses during dispersal which are not included in the original WL model—see detailed explanation below. Please note that for simplicity, and to highlight the main differences between a deterministic all-to-all connected dispersal approximation and probabilistic dispersal (fixed and dynamic networks), we limit our investigations to the case of a meta-population instead of a meta-community, however these concepts can easily be extended to meta-community models. This reduced approach enables us to highlight the main differences following from the extension of a deterministic two patch model/all-to-all connected system to a probabilistic implementation for fixed and dynamic (rewiring) network topologies. We use a directed network in our study to represent the fact that a dispersal/connection event from $i \rightarrow j$ does not imply a reverse dispersal event between $j \rightarrow i$. The modified system equations are:

1 $$\begin{aligned} \begin{aligned} \dot{x_i} = r_i x_i\left( 1-\dfrac{x_i}{K_i}\right) -d\left( x_i-\dfrac{\delta _i}{k_{\mathrm{in}}^{i}}\sum \limits _{j=1}^{N} A_{ij} x_j\right) , i=1, \ldots , N. \end{aligned} \end{aligned}$$

These equations describe a dynamical system $\dot{\mathbf{x }}=(\dot{x}_1,\dot{x}_2,\ldots , \dot{x}_N)^T$ consisting of *N* patches. The first term of the equation represents the logistic dynamics of the local population of species *x* (represented by $x_i$ for the *i*th patch) with a species growth rate $r_i$ and carrying capacity $K_i$ in the *i*th patch, respectively. In the second term, the parameter *d* represents the rate of dispersal for the species, $\delta _i$ represents the efficiency of dispersal, $A_{ij}$ corresponds to the adjacency matrix element containing the unweighted connectivity information for the system, and $k_{\mathrm{in}}^i$ represents the number of incoming connections (in-degree) to patch *i*. For simplicity, we assume that the species growth rate is identical for all patches, hence $r_i=r \; \forall \; i$. We assume that the carrying capacities of the patches are directly proportional to their areas i.e. smaller patches have a lower carrying capacity than the larger ones. The diffusive expression within the brackets corresponds to the difference between the species population in the *i*th patch $x_i$ and the scaled (with $\delta _i$) average input it receives through in-coming connections from other patches via dispersal—this diffusive (interaction) term within brackets is similar to a diffusive local mean-field coupling between connected patches. The dispersal rate parameter *d* can be interpreted as follows: in the absence of internal patch dynamics and any in-coming connections, the linear equation governing the dynamics of $x_i$ is simply, $\dot{x_i} = -d x_i$. Solving this equation gives, $x_i(t)=x_i(0)e^{-d t}$ (*e* being the Euler number), assuming $x_i(0)$ as the initial population of $x_i$ at time $t=0$. Considering a characteristic dispersal time $T_c$ such that for $t=T_c$, the population in the patch exponentially decays by a factor of $e^{-1}$, i.e. $x_i(T_c)=x_i(0)e^{-1}$. Then we can define dispersal rate $d={T_c}^{-1}$, i.e. the inverse of this characteristic dispersal time. The parameter $\delta _i \le 1$ corresponds to the efficiency of dispersal to account for possible losses during the process, since not every individual (here represented in terms of biomass) that is distributed from a patch, will successfully reach another patch. $\delta _i$ can be a function of species characteristics, as well as the local patch environment in general. For the following analysis, we consider this parameter to be identical for the entire meta-population, i.e. $\delta _i=\delta$ $\forall$ *i*. An extreme value of $\delta = 1~(0)$ corresponds to a no (complete) loss scenario. Due to the diffusive nature of inter-patch interaction, the difference between the patch biomass and the loss-modulated (with $\delta$) input received by the patch determines the gradient of diffusion of individuals—into or away from the patch. $A_{ij}$ as mentioned before is the (*i*, *j*)th element of the asymmetric connectivity matrix, which contains the directional connectivity information of the system, with $A_{ii}=0\; \forall \; i$ (no self connections/loops). The normalization (denominator) term $k_{\mathrm{in}}^{i}=\sum \nolimits _{j=1}^{N} A_{ij}$ corresponds to the in-degree of the *i*th patch, i.e. the total number of incoming connections to the patch. The term $A_{ij} = 1 ~(0)$ represents the case when there is a connection/dispersal (no connection/dispersal) from patch *j* $\rightarrow$ *i*. The two extreme cases, (1) $\forall$ $A_{ij}, i \ne j=0$ corresponds to a situation when all patches are isolated and there is an absence of any connections, whereas, (2) $\forall$ $A_{ij}, i\ne j=1$ corresponds to an all-to-all connected system where all patches are sources, as well as sinks to all other remaining patches, implying that the patches are coupled via the overall mean-field. This second case is equivalent to an implicit approach assuming an all-to-all connectivity, which does not take into account the regional distribution of patches and differences in inter-patch distances, and therefore reachability. For $\forall \; A_{ij}, i\ne j=1$ and $\delta = 1$, Eq. (1) reduce to an all-to-all connected system without any dispersal losses, similar to the WL model if reduced to a meta-population.

To estimate the underlying adjacency/connectivity matrix, we implement a probability term where a pair of nodes *i* and *j* are connected with a probability based on (1) Euclidean distance between patches, (2) the size of the patches, as well as (3) the average species dispersal distance. The probability term uses a modified version of the Waxman random graph model^32^. For a regular Waxman random graph model, two nodes *i* and *j* are connected with a probability,

2 $$\begin{aligned} \begin{aligned} P_{ij} =&\beta \exp {\left( \dfrac{-D_{ij}}{\alpha L}\right) }, \qquad \forall (i,j), \qquad i\ne j. \end{aligned} \end{aligned}$$

Here $\alpha$ and $\beta$ $\in (0,1]$ are system specific parameters, $D_{ij}$ is the Euclidean distance between the two nodes, and $L = \mathrm{Max}(D_{ij})\forall (i,j), i\ne j$ corresponds to the maximum inter-patch distance in the system. The parameters $\beta$ and $\alpha$ control the density of connections, and long range connectivity in the system, respectively. For our purpose, we modify Eq. (2) as,

3 $$\begin{aligned} \begin{aligned} P_{ij} = \beta _i \beta _j \exp {\left( \dfrac{-D_{ij}}{\alpha 'L}\right) }, \forall (i,j), i\ne j. \end{aligned} \end{aligned}$$

In Eq. (3), we interpret $\beta$s as normalized patch areas—obtained by dividing each patch area by the largest patch area in the system. As mentioned before, we assume that the patch carrying capacities $K_i \propto \beta _i$ $\forall$ *i*. Consequently, we consider a simple relationship $K_i = \sigma \beta _i$ where the proportionality constant $\sigma =5$ for our analysis. We assume that the probability of dispersal from a source patch *j* to a destination patch *i* is proportional to the normalized areas of both patches, and hence the product term from both the source and sink patch. At the same time, we interpret $\alpha ' \in (0,1]$ as the parameter which determines the dispersal-ability of a species, i.e. the ratio between average distance a species can disperse in the system, and *L*. For our calculations we fix $\alpha '=1$, indicating that the dispersal-ability is equal to the maximum distance *L* in our system—a value of $\alpha ' < 1$ will reduce the dispersal distance accordingly. With this implementation of dispersal probability [Eq. (3)], both patch area and dispersal ability directly affect patch connectivity. For brevity, the modified Waxman network connectivity from Eq. (3) will be referred to as network derived probabilistic connectivity (NPC), and the system Eq. (1) with the NPC adjacency matrix will be referred to as network based model (NBM) henceforth. Furthermore, we refer to model Eq. (1) with a “fixed NPC” as “fixed NBM” and the system with a dynamic “rewiring NPC” as a “rewiring NBM”, respectively. The decision to use either fixed, or dynamic NPCs in models should be based on a comparison between the intrinsic time scales of the species (species specific time scales), and the time scales of changes in the environment (environmental time scales). If the species specific time scales are of the same order, or greater than the environmental time scales, then the dynamic NPC should be used in models. Conversely, if species specific time scales are substantially smaller than the environmental time scales, then a model can utilize a fixed NPC approximation of the inter-patch connectivity. We discuss the methods used for simulation and analysis in the following section. System parameters, network metrics, and their descriptions are briefly summarized in Table 1.

**Table 1:** Parameter summary.

Parameter	Value	Description
$D_{ij}$		Inter-patch distances
*L*	$\text{Max}(D_{ij})$	Maximum inter-patch distance in the system
$\dfrac{D_{ij}}{L}$	$\in (0,1]$	Normalized inter-patch distances
$\beta _i$	$\in [0.3,0.7]$	Normalized patch area: ratio between *i*th patch area and and maximum patch area
$\alpha '$	$=1$	Normalized dispersal–ability: ratio between average species dispersal distance and *L*
*N*	Specified	Number of patches
$r_i$	= 1 $\forall$ *i*	Species growth rate
$K_i$	$\in [1.5,3.5]$	Patch carrying capacity $=\sigma \beta _i$($\sigma =5$)
*d*	$\in [0,20]$	Species dispersal rate
$\delta$	$\in [0,1]$	Dispersal efficiency
$k_{\text{in}}^{i}$	$= \, \sum \limits _{j=1}^{N} A_{ij}$	In-degree for $i^{th}$ patch/node
$C_i$	$= \langle k_{\text{in}}^{i}\rangle$	Ensemble averaged in-degree for *i*th patch/node (“Individual patch and pair-wise patch connectivity” section)
$C_{ij}$		Ensemble averaged pair-wise connectivity (“Individual patch and pair-wise patch connectivity” section)

## Methods

The following section describes the procedures and metrics used for simulation, and the analysis of the model output.

### Generating the connectivity matrix

For NPC related calculations, we first fix a “landscape” using randomly generated areas and inter-patch distances. We further fix the dispersal ability parameter $\alpha '=1$ for generating the NPCs. The landscape and dispersal ability parameters stay fixed for all calculations presented in the manuscript. To estimate the underlying connectivity matrix, we first calculate the probability of connection $P_{ij}$ from Eq. (3), for a pair of nodes *i* and *j* using the system parameters (see Table 1 for ranges). For each pair of nodes, we generate a uniformly distributed random number $\eta$ $\in$ (0, 1) and based on $\eta$, if $\eta < P_{ij}$ ($\eta > P_{ij}$), the adjacency matrix element $A_{ij}=1 (0)$ for dispersal (no dispersal) from $j \rightarrow i$. This process is repeated for all possible $N(N-1)$ pair permutations in the system, providing the asymmetric connectivity matrix representing one possible NPC realization. For calculations of the “fixed NPC” case, several NPC realizations for a fixed landscape and species dispersal ability parameters are generated forming an ensemble, and simulations are repeated for all the NPCs in the ensemble. Similarly for the “rewiring NPC” case, an ensemble of NPC realizations is initially generated. But in this case, each of these realizations serve as the initial NPC which rewires repeatedly after a given number of iterations, also referred to as rewiring time (or time units) during the simulation. At each rewiring event, a new NPC (again with the predefined fixed landscape and dispersal ability parameters) is generated which stays fixed till the next rewiring. This process is repeated for all NPCs in the ensemble.

**Figure 1:**
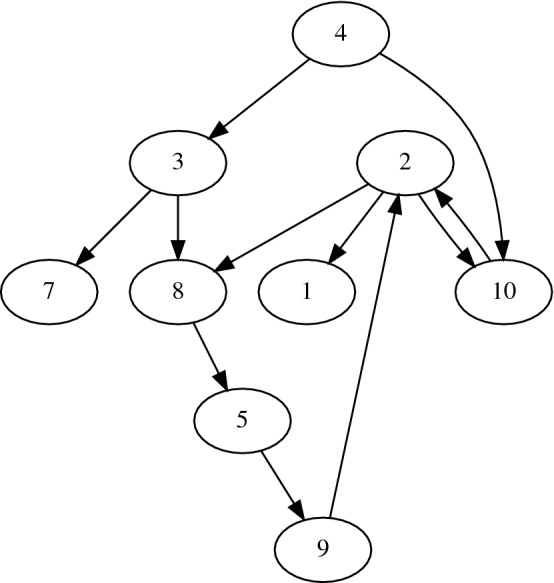
Schematic realization of an NPC consisting of 10 nodes. Connectivity estimated using identical patch areas $\beta _i=0.55 \; \forall \; i$ and an arbitrary underlying landscape with random, normalized inter-patch distances $\dfrac{D_{ij}}{L} \in (0,1]$ $\forall \; i,j$.

As an example, one NPC realization with $N=10$ nodes is shown in Fig. 1. This realization was created for an arbitrary landscape with identical patch-area parameters and randomly generated distances. In this realization, patch 4 and patches 1 and 7 represent two extreme connectivity scenarios. Patch 4 can act only as a dispersal source, since it has only outgoing connections and no incoming connections. Whereas patches 7 and 1 only have incoming connections, and therefore will act as dispersal destinations and never as dispersal sources to other patches. All other patches have incoming as well as outgoing connections and therefore will behave likewise.

### Individual patch and pair-wise patch connectivity

We define $C_i$ to represent the overall patch connectivity for the *i*th patch. Similarly, $C_{ij}$ represents the pair-wise connectivity which gives the likelihood of connection for any two patches *i* and *j* in the system. For the calculation of individual patch connectivity $C_i$, and pair-wise patch connectivity $C_{ij}$, the number of patches is fixed at $N=20$. The ensemble for these calculations consists of 10,000 fixed NPC realizations generated with connectivity matrices for a fixed landscape with randomly generated area and inter-patch distances, and fixed dispersal ability $\alpha '=1$.

1. To explore the influence of patch area on individual patch connectivity, we start by calculating ensemble averaged in (out)-degree $\langle k_{\mathrm{in}}^{i}\rangle$ ($\langle k_{\mathrm{out}}^{i}\rangle$) per node. For this, we generate an ensemble of network realizations as described in “Generating the connectivity matrix” section, and calculate the number of incoming $\langle k_{\mathrm{in}}^{i}\rangle$ and outgoing connections $\langle k_{\mathrm{out}}^{i}\rangle$ for every patch *i* in the network, and for every network realization in the ensemble. We observe that the ensemble size averaged values of in/out connections are almost identical for large ensemble sizes, and therefore use the average in-degree to estimate the individual patch connectivity $C_i$ $\forall$ *i*.
2. For calculating the influence of inter-patch distance on pair-wise patch connectivity, an ensemble of network realizations with identical patch area parameters $\beta _i=\beta$ $(=0.5)$ $\forall$ *i* is generated. Pair-wise connectivity, $C_{ij}$, for a pair of nodes *i*, *j*, located at a distance $D_{ij}$, is then obtained by calculating the ratio between the number of times the pair is connected in the ensemble to the ensemble size. This calculation is repeated for all $N(N-1)/2$ unique pair combinations giving $C_{ij}$ as a function of $D_{ij}$.

### Simulation of system dynamics

In the following (unless stated otherwise), starting from random initial conditions $\forall$ $x_i$ $\in (0,1)$, we simulate the NBMs from Eq. (1), for a total of 3000 time units—30,000 iterations in time steps of $\delta t=0.1$—and ignore the initial 2000 (20,000) time units (iterations) as transients. We use the data from the remaining 1000 (10,000) time units (iterations) for analysis. Deterministic all-to-all connected, and fixed NBM cases are solved using the VODE^33^ solver, whereas the rewiring NBM is solved using the Euler scheme^34^ with a time step $\delta t=0.01$ for error minimization. For the case of homogeneous patches, the carrying capacities (patch areas) are fixed at $K_i=K=2.5$ ($\beta _i=\beta =0.5 \; \forall \; i$), whereas, for the heterogeneous patches (different carrying capacities/patch areas), carrying capacities (areas) are distributed in the range of [1.5, 3.5] ($\beta _i \in [0.3,0.7]$). For calculations with dispersal efficiency $\delta =1$, the dispersal rate $d \in [0,20]$ is increased in steps of 0.1. For two-parameter analysis, $d \in [0,20]$, and $\delta \in [0,1]$ increase in steps of 0.1 and 0.005, respectively.

In our calculations, we assume a population to be extinct when average meta-population biomass $\bar{x}={\mathop {\sum }\nolimits _{i=1}^{N}x_i}/{N} < 0.00001$. We consider an ensemble of $N_{\text{ensemble}}=100$ different network realizations and simulate the dynamics, starting with uniformly distributed random initial conditions $\forall$ $x_i$ $\in (0,1)$, for each *d* value (*d* and $\delta$ values for two-parameter analysis). We note if species exist in the system at the end of the simulation by checking for $\bar{x}>0.00001$ and record these instances for the entire ensemble. From measure theoretic considerations^35^, we use the proportion of ensemble realizations leading to non-zero biomass to the total ensemble size as the persistence probability estimate $P_{\mathrm{per}}$, and the standard error of $P_{\mathrm{per}}$: $\mathrm{SE}(P_{\mathrm{per}})=\dfrac{s_{P_{\mathrm{per}}}}{\sqrt{N_\text{ensemble}}({=}\,10)}$, where $s_{P_{\mathrm{per}}}$ is the ensemble (sample) standard deviation of $P_{\mathrm{per}}$.

## Results and discussion

**Figure 2:**
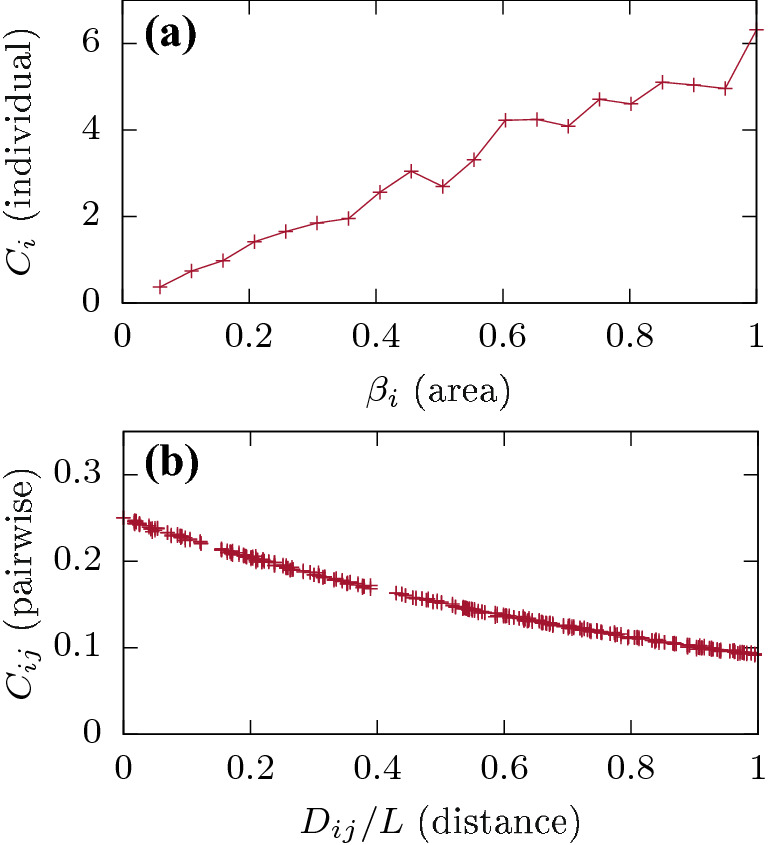
Patch characteristics for NPC with $N=20$. (**a**) individual patch connectivity versus normalized patch area parameter $\beta _i$. (**b**) pair-wise connectivity for the homogeneous patch case with $\beta _i=0.5$ $\forall$ *i*, versus normalized inter-patch distances $\dfrac{D_{ij}}{L}$ $\forall$ $i \ne j$.

In the following section, we start by discussing some important characteristics of NPC. In “Homogeneous patches” section, we look at the system behavior for homogeneous patch parameters and contrast between different connectivity modes, namely (1) all-to-all connected, (2) fixed NBM, and (3) rewiring NBM. We study the homogeneous patch case first to focus on the influence of NPC on system dynamics without any confounding effects of patch heterogeneity. In “Heterogeneous patches” section, we discuss some general results for NBMs with heterogeneous patches, expressed through different patch carrying capacities.

### NPC characteristics

For the case of $N=20$ patches, individual patch $C_i$ and pair-wise patch $C_{ij}$ connectivity estimates are shown as functions of patch areas $\beta _i$ and inter-patch distances $D_{ij}$, in Fig. 2a,b respectively. We observe a nearly linear growth in $C_i$ as a function of patch areas in Fig. 2a, and a nonlinear (exponential) decrease in $C_{ij}$ as a function of inter-patch distances in Fig. 2b. These results nicely illustrate the essential features of the NPC as formulated in Eq. (3): (a) larger patches have more incoming/outgoing connections as compared to smaller patches, and (b) closely located patches connect more frequently as compared to distant ones.

### Homogeneous patches

The influence of dispersal rate *d* on species persistence are investigated and compared for the (1) all-to-all connected model, (2) fixed NBM, and (3) rewiring NBM. For uniformity in comparison, we consider the dispersal efficiency parameter $\delta =1$ in the following “Probability of persistence” and “Influence of patch density and network rewiring rates on persistence” sections.

#### Probability of persistence

**Figure 3:**
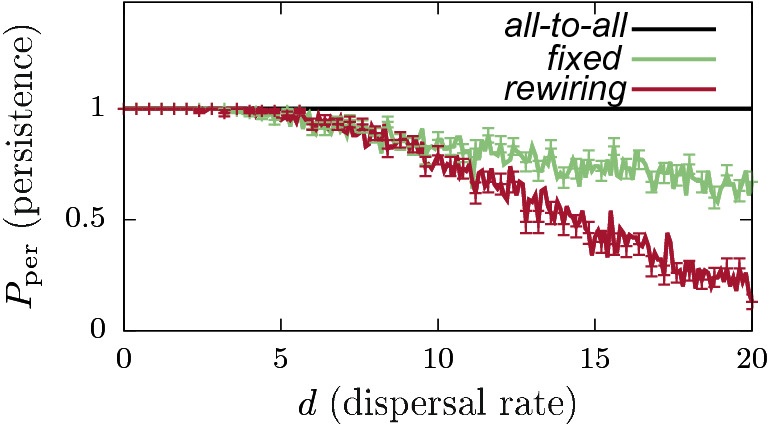
Persistence probabilities $P_{\mathrm{per}}$ as a function of dispersal rate *d*, for different connectivity configurations with $N=20$ and dispersal efficiency $\delta =1$. Curves: all-to-all connected (black), fixed NBM (green), and rewiring NBM (red) (rewiring every 100 time units). The ensemble sizes (number of network realizations) for these calculations are fixed at $N_{\text{ensemble}}=100$ for all three cases. Standard error values $\mathrm{SE}(P_{\mathrm{per}})$, for NBM calculations are highlighted by error bars every four data points to avoid graph overcrowding. Corresponding biomass calculations can be seen in the Online Appendix Fig. A.1.

Figure 3 highlights the influence of NPC on the system dynamics for the $N=20$ case. For the (1) deterministic all-to-all connected case, species persistence $P_{{\rm per}}=1$ in the entire *d* range, and is mathematically expected to stay at the same value for arbitrarily high $d \rightarrow \infty$ values—which is extremely counter intuitive considering the physical bounds and limitations in natural systems. Notably for an all-to-all connected system with $\delta =1$, the second term in Eq. (1) vanishes due to the parameter symmetry and the diffusive mean field nature of the term. Consequently, the dynamics of this system is essentially independent of the dispersal rate *d*, and therefore not affected by any changes in *d*. Hence in this case, local populations in each patch independently follow their logistic growth, and settle on the respective carrying capacity $K_i = K$ $\forall$ *i*—which constitutes the stable equilibrium for the system. On the contrary, for the other two cases of (2) fixed and (3) rewiring NBMs, we observe decreasing $P_{\mathrm{per}}$ estimates for increasing *d* values. These results suggest that several network realizations in the NPC ensembles can lead to species extinction with higher dispersal rates in the NBMs. Furthermore, for rewiring NBM, $P_{\mathrm{per}}$ approaches very low values for higher *d*, before exhibiting species extinction $P_{\mathrm{per}}=0$ for $d \approx 28$ (not shown). On the contrary, we do not observe species extinction $P_{\mathrm{per}}=0$ for quite high *d* values for the fixed NBM case. Please see Online Appendix: Fig. A.1 for the corresponding average biomass calculations.

#### Influence of patch density and network rewiring rates on persistence

**Figure 4:**
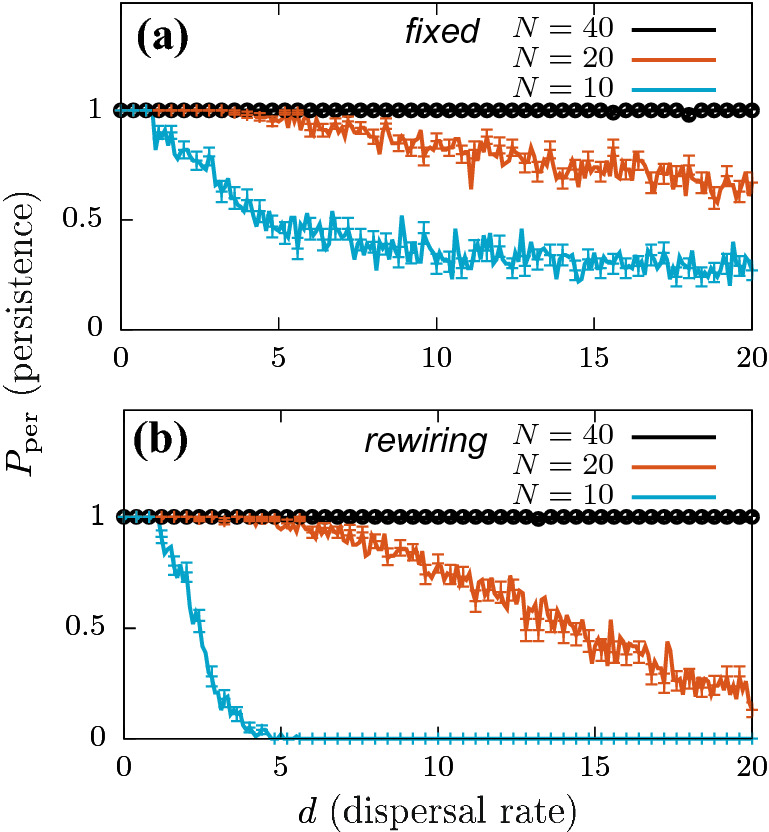
Persistence probabilities $P_{\mathrm{per}}$ as a function of the dispersal rate *d* for (**a**) fixed, and (**b**) rewiring (every 100 time units) NBMs. $N_{\text{ensemble}}=100$ network realizations were used for the simulations, for $N=10$ (blue), $N=20$ (red) and $N=40$ (black) network sizes. Standard error values $\mathrm{SE}(P_{\mathrm{per}})$ for these calculations are highlighted by error bars every four data points. Related biomass calculations can be seen in Online Appendix Fig. A.2.

Figure 4 shows the relationship between species persistence probability $P_{\mathrm{per}}$, and dispersal rate *d* for fixed and rewiring NBMs. Since the value of largest possible distance in the system (*L*) is fixed (fixed landscape) for all calculations, an increase in number of patches *N* corresponds to an increase in patch density in the meta-population. The results suggest that a higher patch density supports species persistence in the system for higher dispersal rates *d*. For $N=10$, $P_{\mathrm{per}} > 0$ (non-zero) for the fixed NBM case over the entire investigated range of *d*, extending to even higher *d* values (not shown here). This implies that there are always some network realizations in the ensemble that support species persistence. In contrast, the species go extinct at $d \approx 5$ for the rewiring NBM case. For $N=20$, both fixed and rewiring NBMs yield non-zero $P_{\mathrm{per}}$ estimates in the investigated *d* range. However we observe a steeper decrease in persistence with increasing *d*, and consequently, extinction for $d \approx 28$ (not shown) for the rewiring NBM. For higher number of patches: $N=40$, $P_{\mathrm{per}} \approx 1$ for the entire *d* range for both fixed and rewiring NBMs, and this behavior extends to even higher *d* values above the range shown in Fig. 4. Our calculations suggest an exponential growth in the total number of connections as a function of number of patches *N* for a fixed landscape, and hence for increasing patch densities (see Online Appendix B, Fig. B.1). Therefore, we can conclude that higher patch densities tend to shift the $P_{\mathrm{per}}$ behavior closer to a highly connected case, which yields $P_{\mathrm{per}}$ results similar to the all-to-all connected situation. Overall, $N=20$ seems to provide an appropriate trade-off between a system which is neither too sparse, nor a densely filled landscape, where a sparse system might lead to a higher proportion of isolated patches, and a dense system can mask the effects of spatially explicit connectivity. Therefore, we use $N=20$ as the standard meta-population size in most of our calculations for the given set of parameter values.

**Figure 5:**
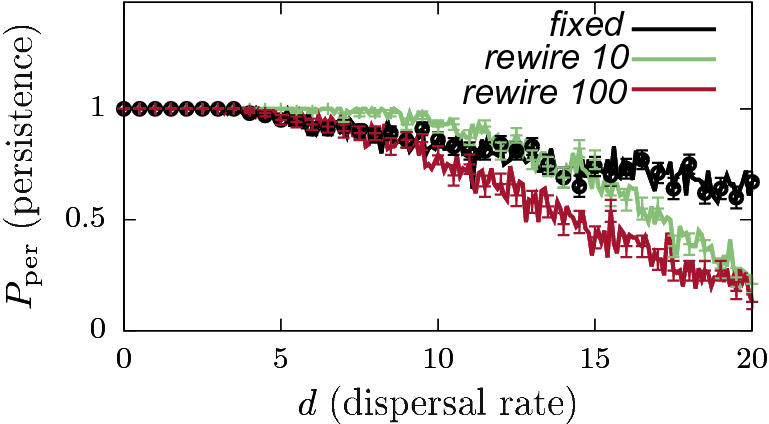
$P_{\mathrm{per}}$ estimates for $N=20$, as a function of dispersal rate *d*, and $\mathrm{SE}(P_{\mathrm{per}})$ are highlighted by error bars every four data points, for identical patches with fixed (black) versus different rewiring rates—every 10 (green) and 100 (red) time units. $N_{\text{ensemble}}=100$ network realizations were used for these calculations. Corresponding biomass estimates are provided in Online Appendix Fig. A.3.

For the case of rewiring NBMs, persistence probability $P_{\mathrm{per}}$ is also influenced by the rewiring time intervals of the connectivity matrix. Results in Fig. 5 compare the $P_{\mathrm{per}}$ estimates for fixed, and rewiring NBMs with different rewiring rates—every 10 and 100 time units. These results show that a faster rewiring NBM promotes species persistence in comparison to the fixed and slower rewiring NBMs, for a range of dispersal rates $d \in (5,10)$—a faster rewiring provides higher $P_{\mathrm{per}}$ estimates in this *d* range. However, the average biomass estimates for all these three cases are quite similar in this dispersal rate range (Online Appendix: Fig. A.3). However, for higher *d* values, species persistence for both rewiring NBMs is lower compared to the fixed NBM. Eventually, the rewiring NBMs exhibit species extinction for higher *d*, whereas, the fixed NBM still yields non-vanishing $P_{\mathrm{per}}$ estimates (not shown).

To better understand the mechanism of species extinction in the meta-population, we need to focus on the critical role of the between-patch interaction (second) term in Eq. (1), i.e. $-d\left( x_i-\dfrac{\delta _i}{k_{\mathrm{in}}^{i}}\sum \nolimits _{j=1}^{N} A_{ij} x_j\right)$. As mentioned before, this term assumes that the dispersal process is diffusive in nature, and the diffusion occurs along a gradient from higher to lower values. With this assumption, the following situations can occur for any $d>0$: (1) average input from other patches is higher than the patch population, i.e. $\dfrac{\delta _i}{k_{\mathrm{in}}^{i}}\sum \nolimits _{j=1}^{N} A_{ij} x_j > x_i$ $\implies$ $d\left( \dfrac{\delta _i}{k_{\mathrm{in}}^{i}}\sum \nolimits _{j=1}^{N} A_{ij} x_j - x_i\right) >0$, the interaction term is positive implying an increase in the number of individuals within the patch via dispersal, i.e. net species movement is directed into the patch due to the population gradient, (2) average input from other patches is less than the patch population, i.e. $x_i >\dfrac{\delta _i}{k_{\mathrm{in}}^{i}}\sum \nolimits _{j=1}^{N} A_{ij} x_j$ $\implies$ $d\left( \dfrac{\delta _i}{k_{\mathrm{in}}^{i}}\sum \nolimits _{j=1}^{N} A_{ij} x_j - x_i\right) <0$, the interaction term is negative implying a decrease in the number of individuals within the patch due to dispersal, i.e. net species movement is directed out of the patch due to the population gradient, (3) no input, i.e. no incoming connections, or an isolated patch case both correspond to an extreme instance of (2), in which case, the interaction term reduces to $- d\left( x_i \right) \le 0$, and (4) input and patch population are identical, i.e. $x_i =\dfrac{\delta _i}{k_{\mathrm{in}}^{i}}\sum \nolimits _{j=1}^{N} A_{ij} x_j$ $\implies$ $d\left( \dfrac{\delta _i}{k_{\mathrm{in}}^{i}}\sum \nolimits _{j=1}^{N} A_{ij} x_j - x_i\right) =0$, consequently the patches effectively decouple due to the absence of a population gradient. Considering these points, the mechanism of an NPC realization leading to species extinction in the corresponding NBM can be interpreted in the following way: for $d=0$, the local populations remain in their respective patches and grow to the respective patch carrying capacity—no dispersal occurs, and therefore no species extinction. For $d>0$, the underlying NPC has a strong influence on the observed dynamics. The underlying connectivity matrix can lead to a situation where some patches have no incoming connections—such a situation is more likely in a sparsely connected network and/or for a network with low patch density. Absence of any incoming connections will lead to case (3) as discussed above, implying to net loss in the local biomass. Consequently, local populations will go extinct in these source-only patches once the dispersal rate is higher than the species growth rate for case (3). This extinction will decrease the biomass flux from these patches to the connected sink patches. With increasing dispersal rates, this will lead to case (2) for these sink patches and eventually they will also experience local extinction, thereby, enabling a cascade which leads to the extinction of the entire meta-population. In terms of bifurcation analysis, this extinction corresponds to a transcritical bifurcation (stability exchange) between equilibria with species persistence and extinction. An important point to consider is that for a fixed landscape, different NPC realizations can give rise to completely different persistence/extinction scenarios. Considering an NPC realization where all patches have at least one incoming connection, and another realization where one/some patches have no incoming connections (source-only case) or are isolated, the described mechanism indicates that species can persist for comparatively higher *d* values in the former case, as compared to the latter.

**Figure 6:**
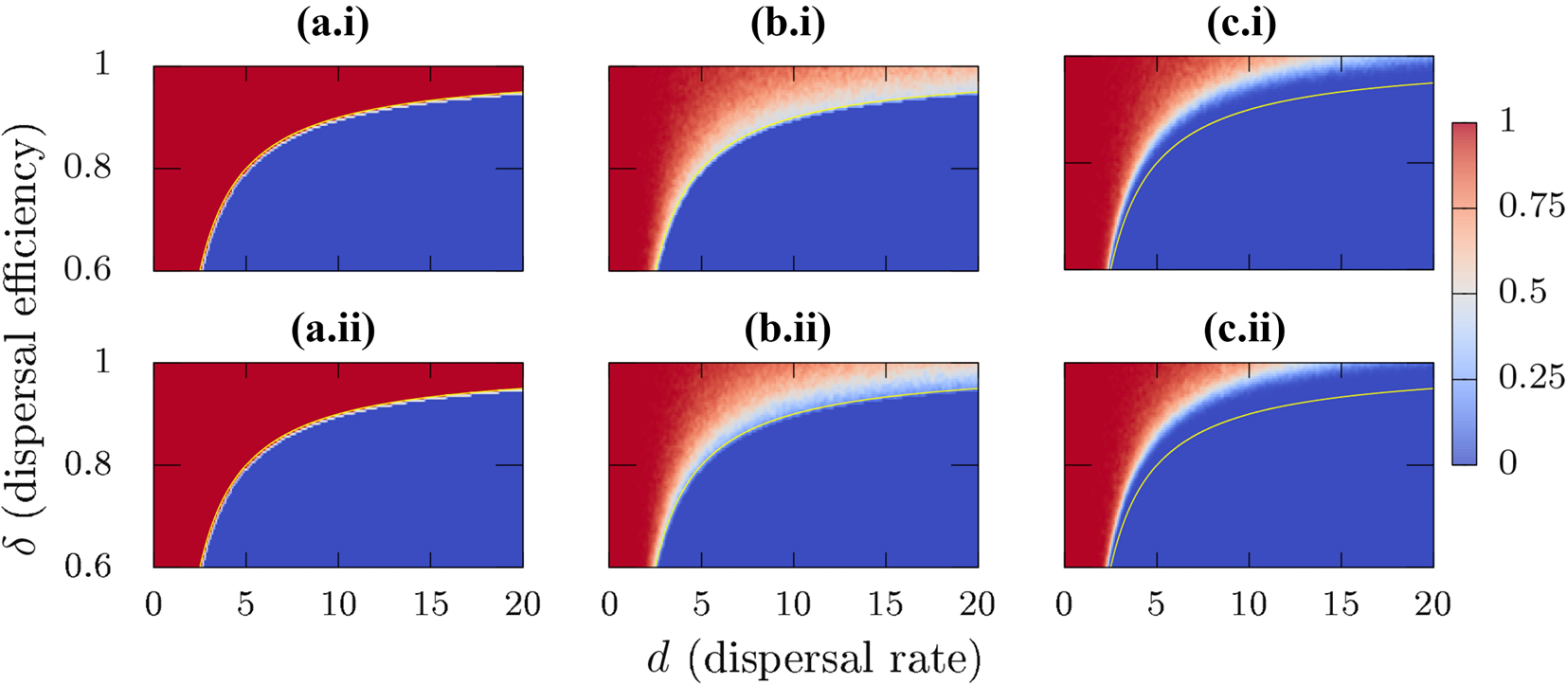
(i) $P_{\mathrm{per}}$ projection on the $(d,\delta )$ plane for homogeneous patches, and (ii) for the heterogeneous patch case. [(**a.i**),(**a.ii**)] correspond to all-to-all connected system, [(**b.i**),(**b.ii**)] to fixed NBM, and [(**c.i**),(**c.ii**)] to rewiring NBM with a rewire every 100 time units. The blue shaded area corresponds to meta-population extinction, i.e. $P_{\mathrm{per}}=0$, whereas red regimes correspond to $P_{\mathrm{per}}=1$. The boundary between persistence and extinction for the all-to-all connected system is indicated by a yellow curve which for comparison, is indicated in all panels. $N_{\text{ensemble}}=100$ for these calculations. Corresponding biomass calculations are shown in Online Appendix Fig. A.4.

**Figure 7:**
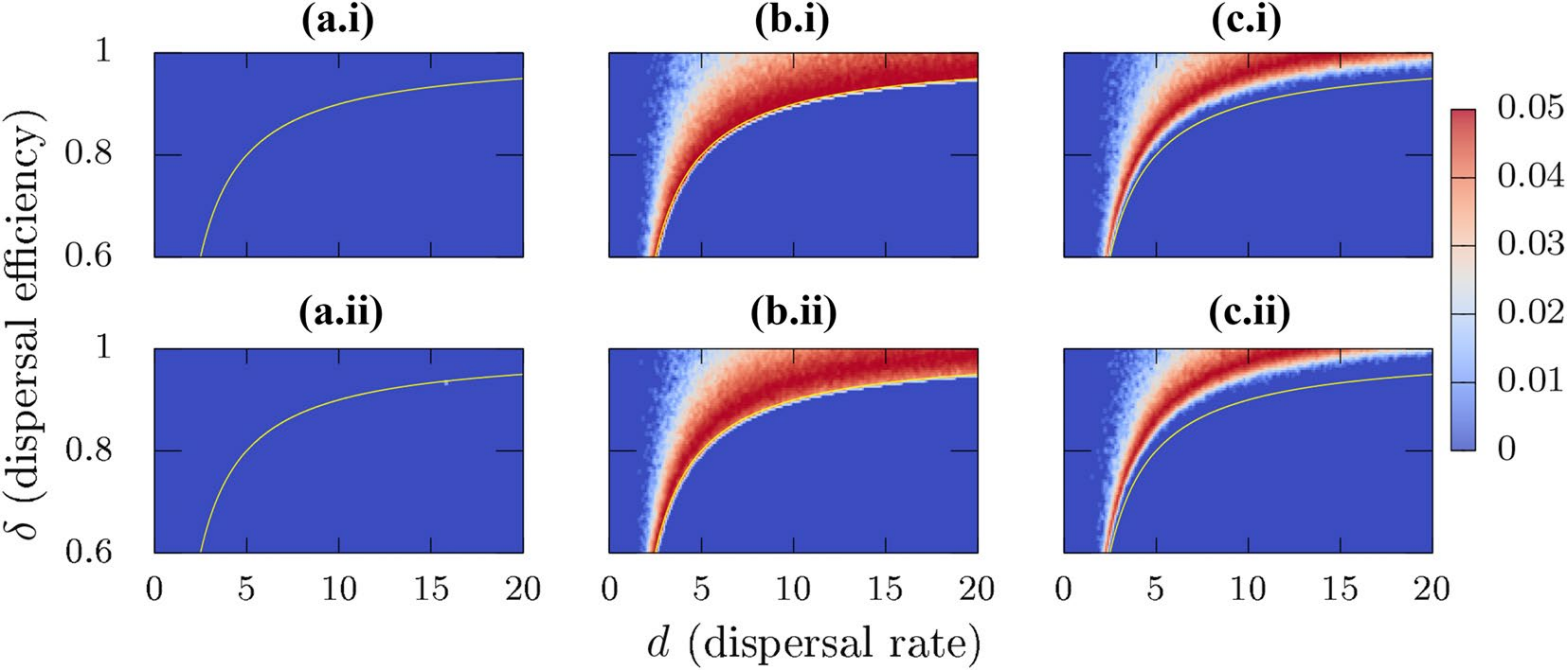
(i) $\mathrm{SE}(P_{\mathrm{per}})$ projection on the $(d,\delta )$ plane for homogeneous patches, and (ii) for the heterogeneous patch case. [(**a.i**),(**a.ii**)] correspond to all-to-all connected system, [(**b.i**),(**b.ii**)] to fixed NBM, and [(**c.i**),(**c.ii**)] to NBM rewiring every 100 time units. The blue shaded area corresponds to regimes where the error is zero. Positive values are highlighted by colors as per the attached color bar. The boundary between persistence and extinction for the all-to-all connected system is again indicated by a yellow curve for comparison in all the panels. $N_{\text{ensemble}}=100$ for these calculations. The dynamical differences between all-to-all connected, and NBMs are even more obvious in these calculations.

#### Dependence of persistence patterns on dispersal rate and efficiency

So far, the results were presented for cases with a dispersal efficiency $\delta _i = \delta = 1$. However, an assumption of 100% efficiency of dispersal is unrealistic. Losses during the dispersal process are likely to occur in natural systems and there is a chance of the species not establishing after arrival in a new patch. To take these losses into account, we introduced $\delta$ in our system Eq. (1). In the following, we investigate species persistence for different connectivity scenarios for $\delta \in [0,1]$, and $d \in [0,20]$. $P_{\mathrm{per}}$ ($\mathrm{SE}(P_{\mathrm{per}})$) behaviors for the homogeneous case are shown in the top row of Fig. 6 (Fig. 7), for (a.i) all-to-all connected, (b.i) fixed NBM, and (c.i) rewiring NBM, respectively.

Starting with the all-to-all connected case, we do not observe any transitions to $P_{\mathrm{per}}=0$ for $\delta =1$. The reason is related to parameter symmetries leading to a vanishing interaction term in Eq. (1) as discussed before. Therefore, the species in their respective patches can grow to the patch carrying capacities $K_i = K$ $\forall$ *i*—see also the average biomass estimates in Online Appendix Fig. A.4. On considering dispersal efficiency $\delta < 1$, the interaction term in Eq. (1) does not vanish, and therefore, species extinction can arise, as seen in Fig. 6(a.i), where red (blue) areas correspond to persistence probabilities of one (zero). Linear stability analysis of the extinction equilibrium, $\mathbf{x} ^*=\mathbf{0} \implies (x_1^*=0,x_2^*=0,\ldots , x_N^*=0)^T$, and any general $N \ge 2$ yields the following eigenspectrum,

4 $$\begin{aligned} \begin{aligned} \lambda _i={\left\{ \begin{array}{ll}(r-d) + d\delta , &{}\quad i=1 (largest) \\ (r-d)-\dfrac{d\delta }{(N-1)} &{}\quad i=2,\ldots ,N. \end{array}\right. } \end{aligned} \end{aligned}$$

Note that these eigenvalues $\lambda _i$s are independent of patch carrying capacities, therefore the persistence–extinction boundary is unaffected by parameter mismatch in the carrying capacities. Using the largest eigenvalue $\lambda _1$, one can follow the transition boundary of the transcritical bifurcation between persistence and extinction in the $(d,\delta )$ plane, which satisfies the expression $\lambda _1 = (r-d) + d\delta =0$. This boundary (yellow curve) is highlighted in Fig. 6(a.i) and is in excellent agreement with the theoretical estimates of this transition boundary (see Online Appendix Fig. A.4 for biomass calculations). Additionally, corresponding $\mathrm{SE}(P_{\mathrm{per}})$ calculations in Fig. 7(a.i) show that the error values for the homogeneous all-to-all connected case are uniformly vanishing in the entire considered $(d,\delta )$ plane. This is due to the behavior of $P_{\mathrm{per}}$ which is 1 before the bifurcation boundary and 0 after the bifurcation leading to meta-population extinction.

For the spatially explicit Fig. 6(b.i) fixed NBM, and Fig. 6(c.i) rewiring NBM cases, we observe that the persistence–extinction transition boundary is not as sharp as for the all-to-all connected case. For comparison, the transition boundary for the all-to-all case is indicated by a yellow curve in these figures. For the fixed NBM case, starting from lower $\delta$ and *d* values, the transition becomes more uncertain as we increase the value of $\delta$—which can be seen by the presence of lighter colored regimes corresponding to low, non-zero persistence probabilities. This ambiguity between persistence and extinction increases even further for higher $\delta$. These results suggest that for high $\delta$, and *d* values, species extinction is possible for the fixed NBM case contrary to the all-to-all connected network, where extinction is impossible in the similar parameter regime. The reasoning behind these results is quite straightforward considering how $P_{\mathrm{per}}$ is estimated. For high $\delta$ and *d* values, there is a proportion of connectivity configurations which lead to species extinction following the mechanism discussed in “Influence of patch density and network rewiring rates on persistence” section. Due to these configurations, we obtain $0< P_{\mathrm{per}} < 1$ in this case. This effect is even more pronounced for the rewiring network, where the blue (extinction) regimes extend to even higher $\delta$ and *d* values, thereby comparatively reducing the regimes of persistence in the parameter space. These results are quite contrary to the all-to-all connected system where the persistence is ensured with $P_{\mathrm{per}} = 1$ along the entire range of *d* values for high $\delta$. The dynamical differences between the all-to-all connected and NBMs are even more conspicuous in the corresponding $\mathrm{SE}(P_{\mathrm{per}})$ calculations, see Fig. 7(b.i),(c.i). For the parameter regimes where $P_{\mathrm{per}} = 1 (0)$, the $\mathrm{SE}(P_{\mathrm{per}})$ values are either zero or very small. For $0< P_{\mathrm{per}} < 1$, $\mathrm{SE}(P_{\mathrm{per}})$ exhibit higher values implying a higher variability in the $P_{\mathrm{per}}$ estimates for NBMs. This comparison also highlights the differences between the fixed and rewiring NBMs. In correspondence to $P_{\mathrm{per}}$ estimates, fixed NBM results exhibit a higher variability for high $\delta$ and high *d* values, as compared to the rewiring NBM case. This is due to the fact that in these ranges of high variability for fixed NBM, the rewiring NBM predicts extinction of the meta-population.

It is quite natural that a meta-population with a local finite growth rate *r* and a biomass loss during dispersal, cannot sustain a population for ever increasing dispersal rates. For lower $\delta$ regimes, all three connectivity scenarios follow this reasoning. For higher $\delta$, the all-to-all connected system can sustain the meta-population for arbitrary high $d \rightarrow \infty$ values with $P_{\mathrm{per}} = 1$. In comparison, our NBM implementations yield reasonable estimates of $P_{\mathrm{per}} < 1$ in high $\delta$ and *d* ranges. Additionally, rewiring NBMs show a higher likelihood of extinction than the fixed NBM—which highlights the fact that, everything else being constant, it is highly likely for a meta-population to exhibit extinction depending on the changes in underlying connectivity alone, which here, correspond to different NPC realizations.

### Heterogeneous patches

Results for the heterogeneous patch case are shown in the bottom row of Fig. 6 for (a.ii) all-to-all connected, (b.ii) fixed NBM, and (c.ii) rewiring NBM. Here we investigate these three configurations for different patch areas $\beta _i$ $\in$ [0.3, 0.7], and consequently different patch carrying capacities, $K_i \in [K_{\min },K_{\max }]$, assigned to the patches in increments of $\left( K_{\max }-K_{\min }\right) /N$. For our calculations in Fig. 6(a.ii),(b.ii),(c.ii), we chose $K_{\min }=1.5$, $K_{\max }=3.5$, and $N=20$. We observe that the results for heterogeneous patches are quite similar to the results with identical carrying capacities. The similarity in the transition boundary can be explained by looking at the eigenspectrum in Eq. (4). The eigenspectrum for the all-to-all connected system is independent of the patch carrying capacities for the extinction equilibrium and therefore, the extinction threshold is not affected by the dissimilarity in the carrying capacities. Accordingly, for the all-to-all connected cases in Fig. 6(a.i),(a.ii), there are no differences, and $P_{\mathrm{per}}$ behaves identically in both the homogeneous [Fig. 6(a.i)] and heterogeneous patch [Fig. 6(a.ii)] cases. Like for the homogeneous case, $\mathrm{SE}(P_{\mathrm{per}})$ calculations in Fig. 7(a.ii) again show that the error values for the heterogeneous all-to-all connected case are uniformly vanishing in the entire considered $(d,\delta )$ plane. For the fixed NPC case [Fig. 6(b.i),(b.ii)], we observe some differences for the transition boundaries for higher $\delta$ values. The $P_{\mathrm{per}}$ estimates at the transition boundaries for high $\delta$ and *d* values are lower (lighter blue dots) in the heterogeneous case [Fig. 6(b.ii)] when compared to the homogeneous case [Fig. 6(b.i)], thereby signifying that in the heterogeneous case, more realizations in the ensemble close to the transition lead to extinction. At the same time, we observe that patch heterogeneity shifts the extinction threshold towards higher $\delta$ values, as compared to the homogeneous case. This implies that for the case of heterogeneous patches, we will observe species extinction for higher $\delta$ values where the homogeneous system still supports persistence. A similar pattern can be observed for the homogeneous [Fig. 6(c.i)] and heterogeneous [Fig. 6(c.ii)] rewiring SEMs. Similar to the homogeneous case, dynamical differences between all-to-all and SEMs are yet again more obvious in $\mathrm{SE}(P_{\mathrm{per}})$ calculations in Fig. 7(b.ii),(c.ii). These observations suggest that unlike in the all-to-all connected case, patch heterogeneity appears to play an essential role in determining the extinction threshold for meta-populations as a function of *d* and $\delta$ in fixed, as well as rewiring NBMs.

## Conclusions and perspectives

In this paper, we introduced our network based probabilistic connectivity (NPC) approach for fixed and rewiring networks to describe species dispersal in the context of meta-populations. We found fundamental differences between the fixed/rewiring network based models (NBMs) and the deterministic all-to-all connected approach. For the case of lossless dispersal ($\delta = 0$), the comparison showed that the probability of species persistence decreases with an increase in the dispersal rate for the NBMs, whereas persistence probabilities were not affected by dispersal rate in case of all-to-all connected systems, even for arbitrarily high dispersal rates. When taking losses during dispersal into account, our results suggest that the NBMs display a smoother transition from persistence to extinction, with increasing dispersal rates for higher dispersal efficiencies, whereas this transition is always discontinuous, forming a sharp boundary between persistence ($P_{per} = 1$) and extinction ($P_{per} = 0$) for the all-to-all connected system. It is important to note that the presented results consider a species which is able to disperse throughout the full landscape , i.e. the dispersal range of the species equals the maximum Euclidean distance in the network. We expect that species with lower dispersal ranges will occupy the system with lower persistence probabilities, and exhibit extinction for a comparatively lower dispersal rate. Moreover, we demonstrated that an all-to-all connected system is obviously unable to capture the influence of patch density on the species extinction threshold, whereas we were able to observe that higher patch densities support species persistence for increasingly higher dispersal rates with the NBM approach. We also observe that for higher patch densities, the behavior of the NBMs tends towards the predictions of an all-to-all connected system. Therefore, the all-to-all connected system can be treated as a limiting case of our NBMs for a patch dense system. Furthermore, our analysis shows that temporal changes in the dispersal patterns, i.e. changes in the inter-patch connectivity over time have an influence on species persistence, and distribution patterns. A detailed study regarding the interplay between different rates of changes in NBMs, and intrinsic species parameters on persistence are beyond the scope of this paper, but will be investigated in subsequent studies. Importantly, the results on species persistence reveal that fixed and rewiring NBMs more realistically capture the dependencies between dispersal rate and dispersal efficiency. The network analysis also reveals that our approach encompasses the exponentially decaying uniform dispersal approach as a long time limit.

The presented NPC formalism enables more flexibility with the temporal resolution, thereby differing from the deterministic approaches, some of which form a special case within the presented framework. The introduced NPC formalism can be adapted to approach various theoretical as well as empirical questions. Some of these research avenues are highlighted in the following. Given that the variability in connectivity on shorter time scales might be highly relevant for projections on changes in species distribution patterns due to climate change and changes in land use patterns, influencing habitat suitability and distribution, our NPCs, and related NBMs allow for the investigation of short term dynamics by incorporating time varying connectivity at different time resolutions into the model. Consequently, on interpreting network ensemble realizations as a result of temporal variations in connectivity, we observe that on shorter time scales, there is a considerable asymmetry between the in- vs. out-degree for the patches, whereas average in-degree vs. out-degree per patch is quite symmetric in the longer time limit. Similarly, sparse meta-populations will have highly asymmetric in-out connections, as compared to dense ones—since the avenues of getting connected in a dense system are higher. Recent studies have explored the relationship between in- and out-degree of a network, using measures like “in-out degree correlation” (IODC), and demonstrated that IODC has a dominant influence over meta-population persistence^30^. It would be interesting to explore these IODC relationships using our approach to explore the generality of these results.

An important future direction of this work will be to study the effects of NPC for meta-communities by extending NBMs to a multi-species system. NPC based meta-community models can foster our understanding on the consequences of the interplay between species interactions and patch connectivity on biodiversity patterns by providing predictions on species distribution patterns^36–38^. Our NPC approach also allows to take into account the local patch quality, thereby enabling predictions regarding species/community persistence under variable patch quality^39,40^. Furthermore, the NPC approach can capture the connectivity characteristics of fragmented landscapes. This can help in simulating the consequences of habitat fragmentation on species distribution and persistence^11,41^ and may serve as a predictive tool for landscape planning and species/biodiversity conservation^42^, as well as for providing projections on corrective measures to avoid species/biodiversity loss—e.g. for the creation of effective habitat corridors^43,44^.

## Supplementary information

Supplementary Information.
