# Supplementary material for: A probabilistic approach to dispersal in spatially explicit meta-populations
Source: Sci Rep. 2020 Dec 17;10:22234. doi: 10.1038/s41598-020-79162-9 (PMC7747636; doi:10.1038/s41598-020-79162-9)
Supplement: Supplementary file 1 — Supplementary Information. [file 41598_2020_79162_MOESM1_ESM.pdf]

A probabilistic approach to dispersal in spatially explicit meta-populations

Appendices

Rajat Karnatak, Sabine Wollrab

Leibniz-Institute of Freshwater Ecology and Inland Fisheries, Müggelseedamnn 310, 12587, Berlin, Germany

Berlin-Brandenburg Institute of Advanced Biodiversity Research (BBIB), 14195 Berlin, Germany

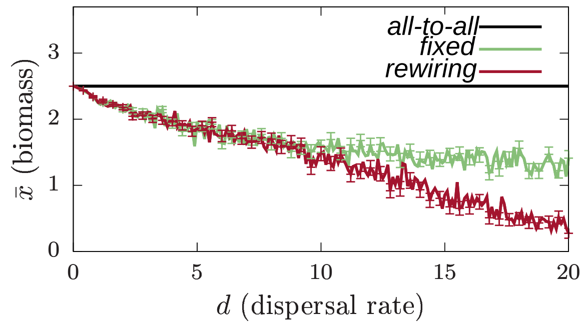

FIG. A.1. Biomass calculation corresponding to Fig. 3: average biomass $\bar{x}$ estimate as a function of dispersal rate d, for different connectivity configurations with $N = 20$ and dispersal efficiency $\delta = 1$. Curves: all–to–all connected (black), fixed SEM (green), and resetting SEM (red) – the underlying network is resetting every 100 time units. $N_{\text{ensemble}} = 100$ for these calculations.

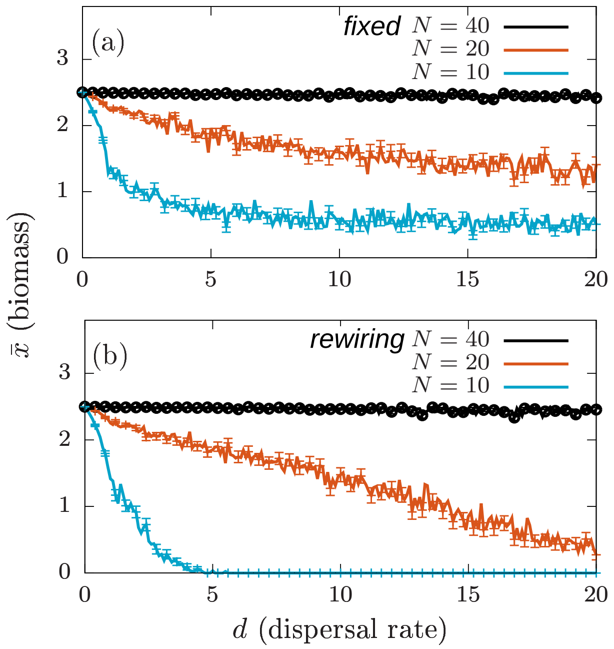

FIG. A.2. Biomass calculation corresponding to Fig. 4: average biomass $\bar{x}$ as a function of dispersal rate d for (a) fixed and (b) resetting SEMs. Ensemble sizes of $N_{\text{ensemble}} = 100$ network realizations were used for these calculations.

Appendix A: Biomass calculations

Biomass calculations corresponding to the persistence probability P_{per} results in Figs. 3, 4, 5, 6 from the main text are presented in this appendix.

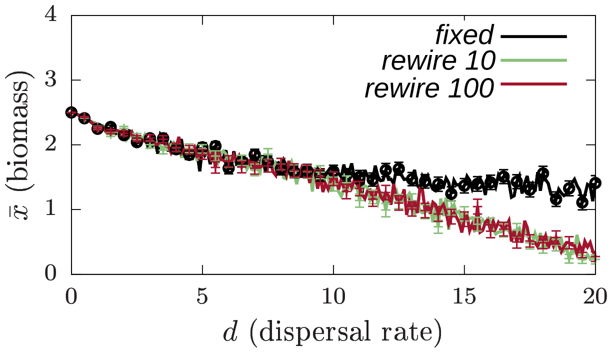

FIG. A.3. Biomass calculation corresponding to Fig. 5: average biomass $\bar{x}$ estimates for $N = 20$, as a function of dispersal rate d for identical patches, and different reset timings. $N_{\text{ensemble}} = 100$ were used for these calculations.

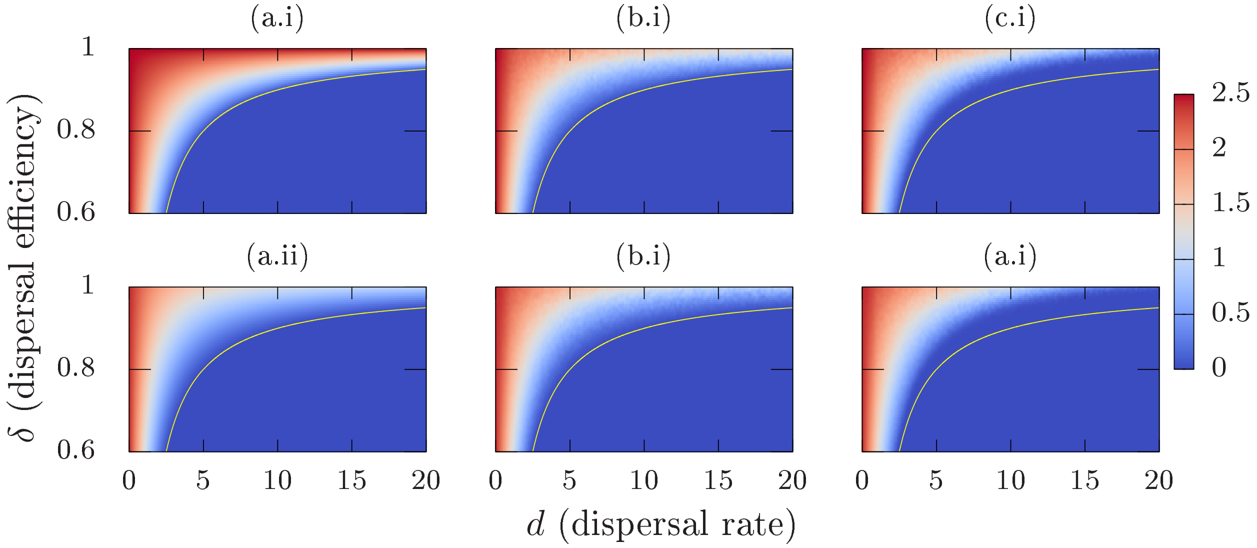

FIG. A.4. Biomass calculation corresponding to Fig. 6: (top row) $\bar{x}$ projection on the (d, δ) plane for homogeneous patches, and (bottom row) for the heterogeneous patch case. Figs. ((a.i), (a.ii)) correspond to all–to–all connected system, ((b.i), (b.ii)) to fixed SEM, and ((c.i), (c.ii)) to SEM resetting every 100 time units. The blue shaded area corresponds to the extinction. The boundary between persistence and extinction for all–to–all connected system is highlighted by a yellow curve in all figures for comparison. $N_{\text{ensemble}} = 100$ for these calculations.

Appendix B: Exponential increase in number of connections

Calculations showing an exponential increase in the total number of connections within the network with an increase in number of nodes (patches) are shown in Fig. B.1.

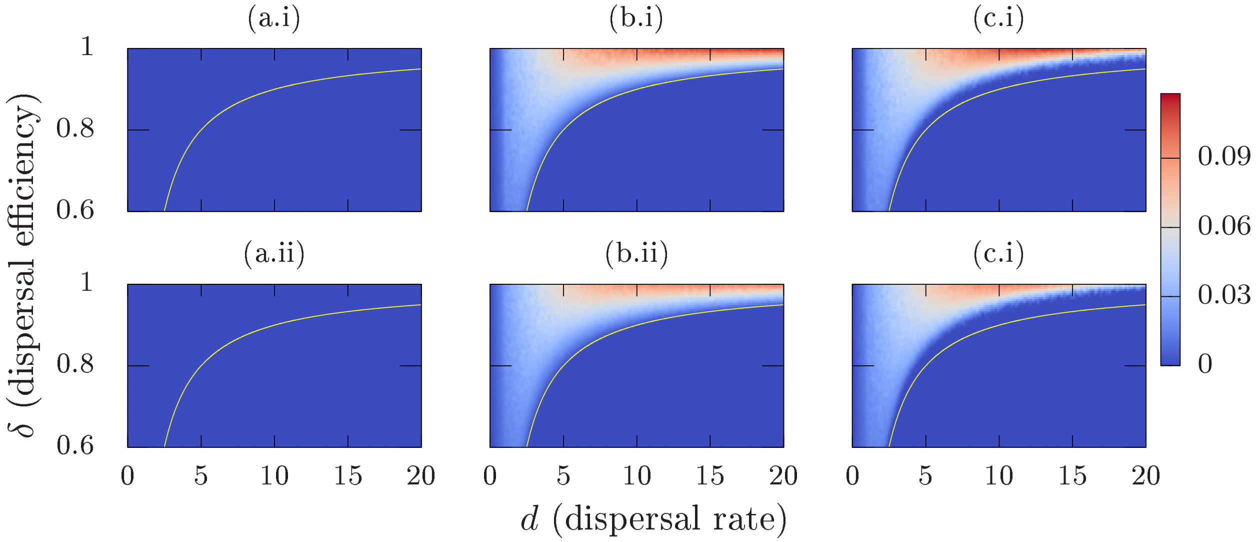

FIG. A.5. (Top) SE($\bar{x}$) projection on the (d, δ) plane for homogeneous patches, and (bottom) for the heterogeneous patch case. Figs. ((a.i), (a.ii)) correspond to all-to-all connected system, ((b.i), (b.ii)) to fixed SEM, and ((c.i), (c.ii)) to SEM resetting every 100 time units. The boundary between persistence and extinction for the all-to-all connected system is again indicated by a yellow curve for comparison in all the figures. $N_{\text{ensemble}} = 100$ for these calculations.

Appendix C: Additional observations

For the logistic patch dynamics, we observe that patches with lower carrying capacities can settle on equilibrium populations larger than their intrinsic carrying capacities for all–to–all connected system and SEMs. Mathematically, these solutions are influenced by the dispersal rate d and the connectivity, and do not exist in absence of dispersal, or do not stabilize for low dispersal efficiency δ values. Dynamically, we observe that smaller patches with a lower carrying capacity receive an overall positive feedback as compared to larger patches. Therefore, the diffusive second term in Eqs. 1 is positive and pushes the patch populations over their carrying capacities, leading to an overshoot. Fig. C.1 highlights these overshoot regimes: Fig. C.1(a), results for the all–to–all connected system are shown: from an ensemble of different initial conditions, we record the proportion of initial conditions from the ensemble leading to an overshoot in any of the patches. For the parameter regimes marked in blue, all initial conditions lead to solutions with no overshoot. On the contrary, parameter regimes in yellow correspond the case where all initial conditions lead to an overshoot in smaller patches.

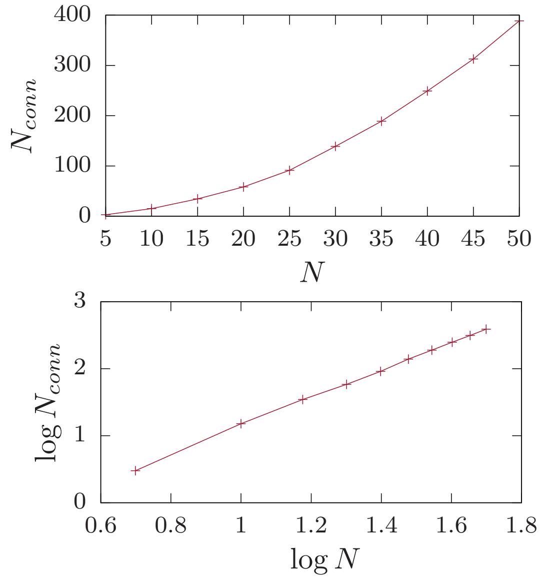

FIG. B.1. (Top): Ensemble averaged total number of connections N_{comm} for networks as a function of the number of nodes N are shown. Values on logarithmic scale (base 10) in the bottom row. Results demonstrate an exponential increase in the number of connections with an increase in the number of nodes. Landscape parameters are fixed for these calculations, and therefore an increase in number of patched implies an increasing patch density. $N_{\text{ensemble}} = 100$ were used for these calculations.

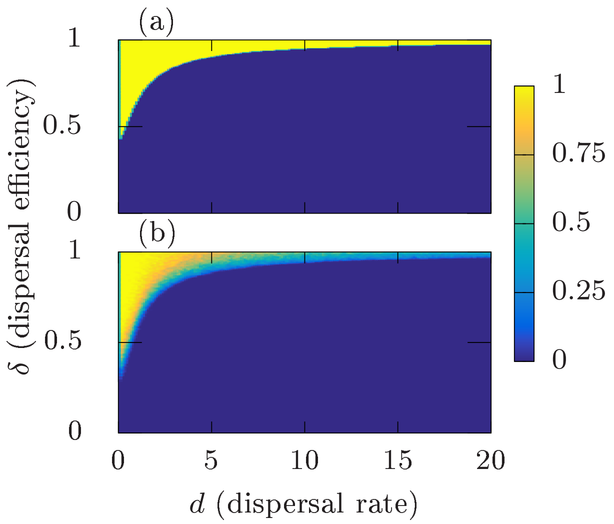

FIG. C.1. (a) Proportion from the ensemble of initial conditions for all–to–all connected, (b) realizations for fixed SEM, leading to the model behavior where low K_i patches settle on $x_i^* > K_i$ equilibrium solutions. Colorbar has been added to aid observation. An ensemble size of 50 was used for these calculations.

Similarly, for the spatially explicit case of fixed SEM, we repeat the same calculation for an ensemble of different SEC realizations. In Fig. C.1(b), we observe that the transition between regimes of overshoot (yellow) and normal dynamics (blue) is not as sharp as in the all–to–all case. The boundary between the white and green parameter regimes is riddled with blue dots which correspond to intermediate values in the overshoot proportion. These intermediate regimes marked by blue dots are less pronounced for $\delta \in (0.5, 1]$ and low d values, but become highly pronounced for $d \in (5, 20]$. The appearance of these blue dots/regimes suggests that for parameter regimes where all–to–all connectivity leads to an overshoot, realizations of the fixed SEC network can still lead to normal solutions. Another important point to note is that higher intrinsic logistic growth rates r_is lead to an expansion in the overshoot regimes in both the cases.
